# Relevance of Non-Bridging Therapy with Heparin during Temporary Interruption of Direct Oral Anticoagulants in Patients with Cancer-Associated Venous Thromboembolism

**DOI:** 10.3400/avd.oa.22-00005

**Published:** 2022-06-25

**Authors:** Takuya Oyakawa, Masafumi Fukumitsu, Aya Ebihara, Taro Shiga

**Affiliations:** 1 Department of Onco-Cardiology/Cardiovascular Medicine, The Cancer Institute Hospital of Japanese Foundation for Cancer Research, Tokyo, Japan; 2 Division of Cardiology, Shizuoka Cancer Center, Sunto-gun, Shizuoka, Japan; 3 Department of Clinical Examination Center, The Cancer Institute Hospital of Japanese Foundation for Cancer Research, Tokyo, Japan

**Keywords:** cancer, venous thromboembolism, heparin bridging, direct oral anticoagulants, cardio oncology

## Abstract

**Objectives:** To evaluate the relevance of non-bridging therapy with unfractionated heparin during the temporary, pre-procedural interruption of direct oral anticoagulants (DOACs) in patients with cancer-associated venous thromboembolism (VTE).

**Materials and Methods:** This retrospective study included 142 patients with cancer and VTE who required temporary interruption of DOACs before invasive procedures. Data, including rates of VTE recurrence, non-major bleeding, and major bleeding, were compared between patients who received or not received alternative therapy with unfractionated heparin during interruption.

**Results:** Sixty-eight patients were prescribed heparin, while 74 were not. There were no differences in age, body mass index, white blood cell count, hemoglobin level, or platelet count between the groups. VTE recurrence was observed in four (6%) and one (1%) patient in the heparin bridging and non-bridging groups, respectively (risk ratio [RR]: 4.4, 95% confidence interval [CI]: 0.50–38.0, p=0.19). Non-major bleeding occurred in three (4%) and two (3%) patients in the bridging and non-bridging groups (RR: 1.6, 95%CI: 0.28–9.48, p=0.67), while major bleeding occurred in 0 (0%) and three patients (4%) (p=0.25), respectively.

**Conclusion:** Our findings confirm the relevance of non-bridging therapy with unfractionated heparin for reducing VTE risk during DOAC interruption in patients with cancer.

## Introduction

Conventionally, bridging with low-molecular-weight heparin or unfractionated heparin is considered when discontinuing antithrombotic drug administration. However, heparin bridging has been regarded as less useful in the recent years. Alternative antiplatelet therapy with heparin is not recommended following coronary intervention, as studies have failed to demonstrate its effectiveness in preventing stent thrombosis.^1)^ In patients with atrial fibrillation, perioperative therapy with heparin as an alternative to warfarin is also not recommended, given that it does not contribute to the reduction of cardiovascular events and may increase the risk of bleeding.^1)^ Moreover, the European Heart Rhythm Association does not recommend perioperative bridging with heparin in patients with atrial fibrillation treated with direct oral anticoagulants (DOACs).^2)^ Further, bridging therapy is associated with an increased risk of bleeding in patients with venous thromboembolism (VTE) during interruption of warfarin therapy for invasive procedures, and it has not been shown to reduce VTE recurrence.^3)^ Periprocedural bridging with heparin is not recommended for patients at low to moderate risk of developing recurrent VTE who require interruption of vitamin K antagonist therapy for invasive procedures.^4)^

Cancer is the most common cause of VTE.^5)^ Indeed, a previous prospective observational study reported a VTE incidence rate of 22.6% among chemotherapy-treated cancer patients.^6)^ Anticoagulant interruption is generally necessary in cancer patients due to surgery, invasive procedures, thrombocytopenia caused by chemotherapy-induced myelosuppression, and cancer progression.^7)^ The rate of VTE recurrence was reported to be higher in cancer patients than in those without cancer.^8)^ Nevertheless, no previous studies have demonstrated the usefulness of bridging with heparin when interrupting DOAC therapy in cancer patients who develop VTE. Therefore, in the present study, we aimed to examine the relevance of non-bridging therapy with unfractionated heparin during temporary interruption of DOACs for VTE treatment in cancer patients.

## Materials and Methods

**Figure 1:**
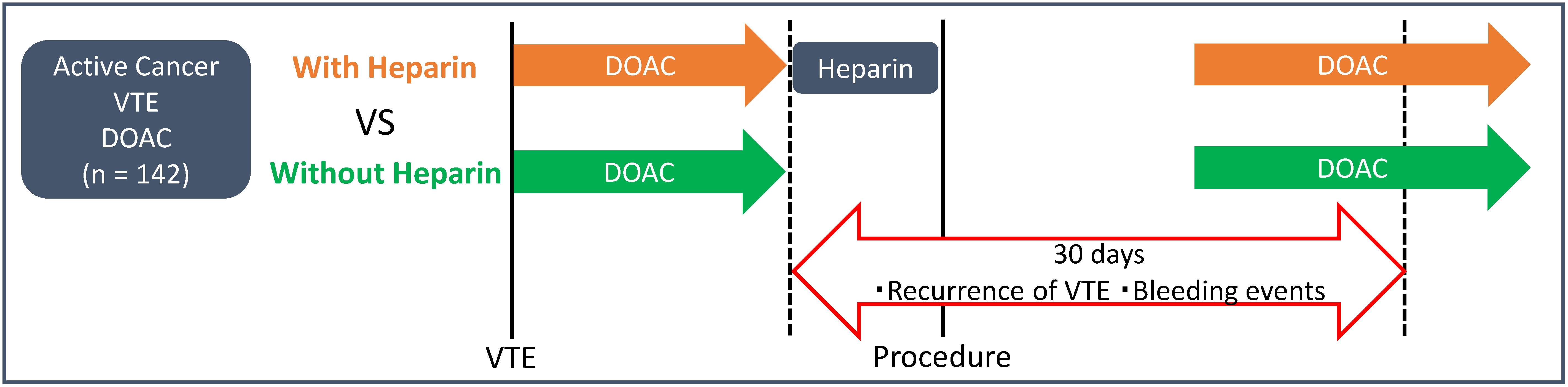
Fig. 1 Methods.

VTE: venous thromboembolism; DOAC: direct oral anticoagulant

Of the 300 consecutive patients undergoing DOAC treatment for cancer-associated VTE at our hospital between January and October 2018, those in whom temporary interruption of DOAC treatment was required prior to invasive procedures were included in this study. Cancer patients were defined as those diagnosed with cancer within the last 6 months or those diagnosed with recurrent, regionally advanced, or metastatic cancer. We retrospectively compared patients who received alternative therapy with unfractionated heparin and those who received no alternative therapy during the temporary interruption period. Unfractionated heparin was used as an alternative therapy because low-molecular-weight heparin is not approved for VTE in Japan. The presence or absence of unfractionated heparin bridging was determined by the treatment policy of the attending physician for cancer management. We confirmed the incidence of VTE recurrence, non-major bleeding, and major bleeding during the evaluation period.^9,10)^ Similar to its definition in previous studies, the evaluation period in this study was defined as 30 d after the first day of DOAC interruption^11)^ (**Fig. 1**). Perioperative patients used intermittent pneumatic compression devices and elastic stockings.

We collected data concerning the following: age, sex, performance status (Eastern Cooperative Oncology Group), body weight, body mass index, creatinine, platelet count, hemoglobin, pulmonary thromboembolism, types of anticoagulant drugs used before the procedure, types of cancer, cause of DOAC interruption, duration from diagnosis of VTE to discontinuation of DOAC due to the procedure, types of anticoagulant drugs used after the procedure, duration of the anticoagulant interruption period, recurrence of VTE, major bleeding, and non-major bleeding.

Wilcoxon’s rank-sum test or Fisher’s exact test was used to make comparisons between the two groups and calculate the risk ratio. The Kaplan–Meier method was used for the analysis of the duration difference of the median time from diagnosis of VTE to discontinuation of DOAC and the duration of the anticoagulant interruption period, as well as to calculate the 95% confidence intervals (CIs). Moreover, the log-rank test was used to compare the two groups. Statistical significance was set at p<0.05. Data were analyzed using JMP9 software (SAS Institute Inc., Cary, NC, USA).

This study was approved by our Institutional Review Board (2020-GA-1073). Informed consent was obtained by opt-out methodology.

## Results

### Patients

Of the 300 consecutive patients undergoing DOAC treatment for VTE, 142 required a temporary interruption of DOAC therapy prior to invasive procedures. Sixty-eight patients received alternative therapy with unfractionated heparin, while 74 patients did not receive alternative therapy. The characteristics of the included patients are shown in **Table 1**. In terms of patient background, there were statistically significant differences in the creatinine levels and causes of DOAC interruption between the two groups. The mean creatinine level was 0.69 mg/dL in the heparin bridging group and 0.75 mg/dL in the non-bridging group. When the causes of DOAC interruption were divided into surgical and non-surgical categories, the number of patients who underwent heparin bridging was higher among those in whom DOAC was interrupted due to surgery than among those in whom treatment was interrupted for other reasons, which included invasive treatment and endoscopic therapy. The median durations from the diagnosis of VTE to the discontinuation of DOAC due to the procedure were 12 (95%CI: 8–17, interquartile range [IQR]: 5–28) and 25 (95%CI: 18–35, IQR: 12–51) days in the heparin bridging and non-bridging groups, respectively; thus, the duration was significantly shorter in the heparin bridging group than in the non-bridging group (p<0.01).

**Table 1:** Table 1 Patient characteristics

		With UFH (n=68)		Without UFH (n=74)		p-value
Age	Median, years	70		69		0.85
Sex	Male	20		33		0.08
Performance status	0/1	65		74		0.11
	2/3/4	3		0		
Body weight	Median (IQR) kg	50.8 (45.9–59.1)		55.1 (46.5–66.9)		0.13
Body mass index	Median (IQR) kg/m^2^	21.2 (18.4–22.6)		21.5 (19.2–24.5)		0.25
Creatinine	Median (IQR) mg/dL	0.66 (0.54–0.77)		0.71 (0.61–0.87)		0.02
Platelet count	Median (IQR) 10^4^/L	21.9 (18.0–28.8)		23.5 (18.2–26.8)		0.99
Hemoglobin	Median (IQR) g/dL	12.6 (10.9–13.5)		11.9 (10.6–13.4)		0.22
Proximal DVT	Yes	9		13		0.50
Pulmonary thromboembolism	Yes	5		8		0.57
Anticoagulant drug	Edoxaban	36		36		—
	Apixaban	28		33		
	Rivaroxaban	4		5		
Type of cancer		Ovarian cancer	14	Colorectal cancer	15	—
		Gastric cancer	10	Gastric cancer	11	
		Uterine cancer	8	Uterine cancer	9	
Cause of DOAC interruption	Operation	62		54		0.01
	Other	6		20		

Abbreviations: DOAC: direct oral anticoagulant; UFH: unfractionated heparin; IQR: interquartile range; DVT: deep vein thrombosis

### DOAC interruption period

**Figure 2:**
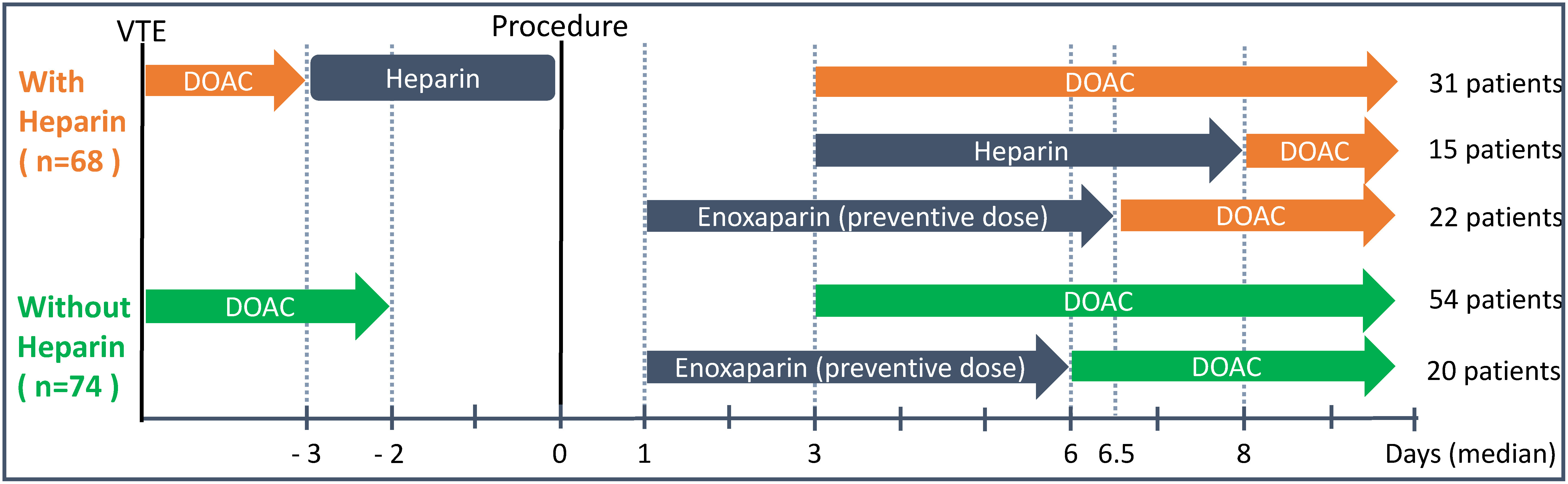
Fig. 2 Anticoagulant interruption.

VTE: venous thromboembolism; DOAC: direct oral anticoagulant

The clinical course of the DOAC interruption period is presented in **Fig. 2**. In the bridging group, the median period of DOAC interruption before the procedure was 3 (95%CI: 2–3) days. After the procedure, anticoagulant treatment was resumed in this group using DOACs, unfractionated heparin followed by a change to DOACs, or a preventive dose of enoxaparin followed by a change to DOAC. In the non-bridging group, DOAC was stopped at a median of 2 (95%CI: not estimated) days before the procedure, and treatment was resumed following the procedure using either DOACs or a preventive dose of enoxaparin followed by a change to DOACs. When the total period of anticoagulant interruption was compared between the groups, the median duration of interruption was significantly longer in the non-bridging (4 days, 95%CI: 3–6 days) than in the bridging group (2 days, 95%CI: 1–3 days). Whether patients treated with enoxaparin were included or excluded, the duration of DOAC interruption was significantly longer in the heparin bridging group than in the non-bridging group.

### VTE recurrence and bleeding events

VTE recurrence was observed in four patients (6%) in the heparin bridging group and in one patient (1%) in the non-bridging group. There were no significant differences between the two groups (risk ratio [RR]: 4.4, 95%CI: 0.50–38.0, p=0.19) (**Table 2**). Non-major bleeding occurred in three (4%) and two (3%) patients in the bridging and non-bridging groups, respectively (RR: 1.6, 95%CI: 0.28–9.48, p=0.67). Major bleeding occurred in 0 patients in the bridging group and in three (4%) patients in the non-bridging group (p=0.25). Thus, there was also no significant difference in bleeding between the two groups. The results were similar whether patients treated with enoxaparin were included or excluded (**Table 3**).

**Table 2:** Table 2 Venous thromboembolism recurrence and bleeding events

	With UFH (n=68)	Without UFH (n=74)	RR (95%CI)	p-value
Recurrence of VTE	4 (6%)	1 (1%)	4.4 (0.50–38.0)	0.19
Non-major bleeding	3 (4%)	2 (3%)	1.6 (0.28–9.48)	0.67
Major bleeding	0 (0%)	3 (4%)	NE	0.25

Abbreviations: UFH: unfractionated heparin; RR: risk ratio; CI: confidence interval; VTE: venous thromboembolism; NE: not estimated

**Table 3:** Table 3 Venous thromboembolism recurrence and bleeding events (excluding patients treated with enoxaparin)

	With UFH (n=46)	Without UFH (n=54)	RR (95%CI)	p-value
Recurrence of VTE	3 (7%)	1 (2%)	3.5 (0.38–32.7)	0.33
Non-major bleeding	2 (4%)	2 (4%)	1.2 (0.17–8.0)	1.00
Major bleeding	0 (0%)	3 (6%)	NE	0.25

Abbreviations: UFH: unfractionated heparin; RR: risk ratio; CI: confidence interval; VTE: venous thromboembolism; NE: not estimated

## Discussion

In this study, we investigated the significance of heparin bridging therapy during temporary interruption of DOAC treatment for VTE in cancer patients. Our findings indicate that bridging therapy with unfractionated heparin during the DOAC interruption period did not reduce the rate of VTE recurrence.

In Europe, heparin bridging during DOAC interruption has been reported in numerous patients, including those with various indications for DOAC (i.e., 81% of patients with atrial fibrillation and 22% of patients with cancer); however, the results showed that heparin bridging increased the incidence of major bleeding without reducing the rate of thromboembolic events.^12)^ Our study also showed no reduction in the occurrence of thromboembolism (i.e., our results on VTE recurrence are in accordance with those of the previous European study). Therefore, heparin bridging is not likely to be useful in VTE treated with DOAC in Japanese cancer patients.

Warfarin has long been used for oral anticoagulant therapy. A period of 5–7 days is required for the anticoagulant effects of warfarin to appear/disappear.^13)^ Thus, heparin is used during warfarin interruption, given the long discontinuation period for warfarin therapy. A previous study reported that when warfarin treatment was interrupted in patients with atrial fibrillation prior to invasive procedures, the mean discontinuation period was 5.3 days before the procedure, and treatment was resumed within a mean of 1.4 days after the procedure.^11)^ The authors further reported that heparin bridging increased bleeding without reducing the rate of thromboembolic events. Another study reported that the median duration of DOAC interruption was 2 days before the procedure and 1 day after the procedure.^12)^ As this suggests that a shorter discontinuation period is required for DOACs than for warfarin, thrombotic events may be less likely to occur in patients treated with DOACs than in those treated with warfarin. Indeed, our findings indicate that there was no difference in the rate of thrombotic events between the heparin bridging and non-bridging groups in our study.

In this study, the median period of anticoagulant interruption was 4 days in patients without heparin bridging and 2 days in patients with heparin bridging. This short difference in duration may explain in part why there was no increase in bleeding events after heparin bridging.

Although Kuo et al. included patients with various indications for anticoagulation, a previous meta-analysis examined data for patients undergoing heparin bridging during warfarin or DOAC interruption.^14)^ The meta-analysis included six randomized controlled trials and 12 cohort studies. When the randomized controlled trials were considered, there was no difference in the rate of thromboembolic events between the bridging and non-bridging groups, and the results were characterized by low heterogeneity. The combined analysis of randomized controlled trials and cohort studies also indicated that heparin bridging did not reduce the occurrence of thromboembolism. There was no report supporting that heparin bridging reduced thromboembolism events in these 18 studies included in the meta-analysis. In contrast, heparin bridging significantly increased both the major bleeding and overall bleeding events. At least in terms of thromboembolic events, our findings are consistent with those of this meta-analysis for warfarin or DOAC.

This study had some limitations, including its small sample size. The statistical power of this study was calculated to be 64%, assuming that the error rate was 10% on one side.^15)^ Larger prospective studies are required to verify the relevance of non-bridging therapy with heparin in patients with cancer and VTE who require DOAC interruption prior to invasive procedures. Second, the risk of VTE differs depending on the type of cancer. Therefore, future studies should assess the effect of cancer type on the outcomes of heparin bridging in patients with VTE.

## Conclusion

Based on our findings, heparin bridging may not be useful during DOAC interruption in patients with cancer and VTE, in accordance with findings reported for anticoagulant interruption in patients with atrial fibrillation and in those for antiplatelet interruption following coronary intervention. Although heparin bridging has been conventionally used since the warfarin era, our findings suggest that the requirement for this strategy should be reconsidered when DOAC treatment must be interrupted in patients with VTE.
